# Transcriptomic and Metagenomic Biomarkers in Peri-Implantitis: A Systematic Review, Diagnostic Meta-Analysis, and Functional Meta-Synthesis

**DOI:** 10.3390/medsci13030187

**Published:** 2025-09-12

**Authors:** Carlos M. Ardila, Eliana Pineda-Vélez, Anny M. Vivares-Builes

**Affiliations:** 1 Department of Periodontics, Saveetha Dental College and Hospitals, Saveetha Institute of Medical and Technical Sciences, Saveetha University, Saveetha 600077, India; 2 Biomedical Stomatology Research Group, Basic Sciences Department, Faculty of Dentistry, Universidad de Antioquia U de A, Medellín 050010, Colombia; eliana.pineda@uam.edu.co (E.P.-V.); anny.vivares@uam.edu.co (A.M.V.-B.); 3 Faculty of Dentistry, Institución Universitaria Visión de las Américas, Medellín 050040, Colombia

**Keywords:** peri-implantitis, biomarkers, transcriptomics, machine learning, bioinformatics, meta-analysis, immune profiling

## Abstract

**Background/Objectives:** Evidence from transcriptomic and histopathologic studies has revealed that peri-implantitis lesions are characterized by deeper inflammatory infiltration, increased immune cell accumulation, and distinctive molecular signatures. This systematic review aimed to evaluate the diagnostic and pathophysiological potential of transcriptomic, metagenomic, and bioinformatic biomarkers in peri-implantitis by integrating findings from bioinformatics and machine learning-based studies. The dual objective was to identify biologically relevant markers and assess the accuracy of predictive models, addressing diagnostic gaps in peri-implant disease management. **Methods:** Eligible designs included cross-sectional, case–control, and cohort studies. Literature searches were conducted across PubMed, EMBASE, Scielo, and Scopus, with independent screening, data extraction, and quality assessment. Functional meta-synthesis was used to thematically organize biomarkers and pathways, while diagnostic meta-analysis pooled ROC-AUC values to assess model performance. **Results:** Eleven studies met the inclusion criteria. Functional synthesis revealed five recurring biomarker themes: innate and adaptive immune responses, immune cell infiltration, fibroblast activation, and ceRNA regulation. A meta-analysis of six studies reported a pooled AUC of 0.91 (95% CI: 0.88–0.93) with I^2^ = 0%, indicating no heterogeneity, supporting the reliability of ML-based models in distinguishing peri-implantitis from healthy conditions. Sources of variation included differences in validation strategies and data preprocessing. **Conclusions:** Integrating transcriptomic, metagenomic, and bioinformatic biomarkers with machine learning may enable earlier and more accurate diagnosis of peri-implantitis. The identified biomarkers highlight molecular and microbial pathways linked to inflammation and tissue remodeling, underscoring their potential as diagnostic indicators and therapeutic targets with translational relevance.

## 1. Introduction

Peri-implantitis is defined as a pathological condition characterized by inflammation in the peri-implant mucosa and progressive loss of supporting bone [1]. The 2017 World Workshop on the Classification of Periodontal and Peri-Implant Diseases and Conditions classified peri-implantitis as a plaque-associated condition occurring in patients with dental implants that have achieved osseointegration. Clinically, it presents with bleeding on probing, increased probing depths, and radiographic bone loss [2]. Its reported prevalence varies across studies, with estimates ranging from 22% to 56.6% at the patient level [3].

Although the clinical management of peri-implantitis often follows treatment principles derived from periodontitis, the biological behavior of these diseases differs significantly. Transcriptomic and histopathologic investigations have identified deeper inflammatory infiltrates, higher immune cell densities, and distinct molecular patterns in peri-implantitis lesions, suggesting a unique pathophysiological profile [4,5,6]. These findings highlight the need for a tailored understanding of peri-implant disease beyond conventional diagnostic frameworks.

Traditional diagnostic approaches, primarily based on clinical and radiographic assessments, provide limited insights into the molecular complexity driving peri-implant tissue destruction [7]. In recent years, the application of omics technologies—particularly transcriptomics and metagenomics—combined with computational methods such as machine learning (ML) and bioinformatics, has enabled the identification of biomarkers with diagnostic and prognostic value. Studies have identified key differentially expressed genes (e.g., *TLR4*, *CXCL8*, *FLT3*, and *IL1B*), immune cell infiltration profiles (e.g., CD4+ T cells and macrophages), and microbiome signatures (e.g., distinct *Fusobacterium nucleatum* clades) in peri-implantitis [8,9,10].

Despite the growing volume of research, there remains a lack of integrated synthesis evaluating the role of biomarkers derived from these multi-omics platforms using standardized methodological frameworks. Existing investigations often focus on isolated analytical strategies or restricted sample types, and do not comprehensively examine the functional relevance of the findings or their potential translational applications. Furthermore, diagnostic performance metrics—such as area under the receiver operating characteristic curve (AUC) values—are rarely synthesized across machine learning (ML) studies, limiting the development of robust predictive tools for clinical use [11].

This study addresses this gap through a dual approach: a functional meta-synthesis of biomarkers and immune-microbial pathways based on transcriptomic and bioinformatic evidence, and a diagnostic meta-analysis of ML models reporting performance metrics such as the receiver operating characteristic–area under the curve (ROC-AUC). This integrative strategy aims to reveal converging biomolecular themes, identify robust diagnostic biomarkers, and assess methodological strengths and limitations in current computational studies on peri-implantitis.

Therefore, the objective of this study is to systematically synthesize the evidence on transcriptomic, metagenomic, and bioinformatic biomarkers identified through advanced computational and machine learning approaches in peri-implantitis and to evaluate the diagnostic accuracy of ML-based models. The ultimate aim is to support the development of more precise diagnostic tools and to enhance understanding of peri-implantitis pathophysiology through functional biomarker mapping.

## 2. Materials and Methods

### 2.1. Protocol and Registration

This systematic review was conducted in accordance with the Preferred Reporting Items for Systematic Reviews and Meta-Analyses (PRISMA 2020) guidelines [12]. The protocol was prospectively registered in the International Prospective Register of Systematic Reviews (PROSPERO database CRD420251114576).

### 2.2. Eligibility Criteria

Studies were considered eligible if they were original research articles with a cross-sectional, case–control, or cohort design, and if they applied transcriptomic, metagenomic, or bioinformatic methodologies to investigate biomarkers associated with peri-implantitis. Eligible studies had to include human-derived peri-implant tissue samples—such as gingival, submucosal, or crevicular tissues—and report on transcriptomic, microbial, or bioinformatic biomarkers. Additionally, studies that employed machine learning or statistical feature selection techniques (e.g., LASSO, SVM-RFE, Boruta) for biomarker identification were included. Studies were excluded if they were reviews, editorials, commentaries, conference abstracts without full text, animal or in vitro experiments, or lacked a clear bioinformatics or machine learning component.

### 2.3. PICO Framework

The present systematic review was structured according to the PICO framework to guide eligibility criteria and the synthesis process:

- Population (P): Human-derived peri-implant tissue samples affected by peri-implantitis.
- Intervention (I): Application of transcriptomic, metagenomic, or machine learning methods for biomarker discovery.
- Comparator (C): Healthy peri-implant or periodontal tissues in matched or comparative analyses.
- Outcomes (O): Identification of key transcriptomic, microbial, or bioinformatic biomarkers as the primary outcome, and diagnostic performance metrics (e.g., ROC-AUC), methodological strategies, pathway classification, and immune cell deconvolution as secondary outcomes.

### 2.4. Information Sources and Search Strategy

A comprehensive literature search was performed in PubMed/MEDLINE, EMBASE, SciELO, and Scopus for studies published until July 2025. No start date or language restrictions were applied. The search strategy combined controlled vocabulary and free-text terms using Boolean operators. Core terms included “peri-implantitis” or “periimplantitis” combined with bioinformatics or related computational biology, machine learning, artificial intelligence, RNA-seq, gene expression, WGCNA, ceRNA, transcriptome, and metagenomics, together with outcome-related terms such as biomarkers, immune signatures, differential expression, microbiome, and microbiota. To improve recall, additional computational deconvolution approaches (e.g., CIBERSORT and related methods) were also incorporated. In PubMed, a standard filter was applied to exclude animal-only studies while retaining all human studies. The full database-specific search strategies are provided in Supplementary Table S1.

Additionally, the reference lists of all included studies were manually screened to identify further relevant publications.

### 2.5. Study Selection

All retrieved citations were imported into EndNote for de-duplication. Two reviewers independently screened the titles and abstracts of the articles, followed by full-text assessments to determine eligibility. Any discrepancies were resolved by discussion, and if needed, arbitration by a third reviewer was employed to reach consensus.

### 2.6. Data Extraction

Data were extracted independently by two reviewers using a standardized and pre-tested form. Extracted data included study metadata (authors, year, country, study design), sample characteristics, tissue type, transcriptomic platform (e.g., RNA-seq, microarray, metagenomics), biomarker discovery strategies (e.g., differential expression analysis, network construction, ML-based feature selection), bioinformatic tools applied, and the nature of the reported biomarkers. Details of validation methods—such as qPCR, ROC analysis, or external dataset replication—were also collected. Discrepancies in data extraction were resolved through consensus or by consulting a third reviewer.

### 2.7. Outcome Measures

The primary outcome was the identification of transcriptomic, immunological, or microbial biomarkers associated with peri-implantitis. Secondary outcomes included diagnostic performance metrics such as ROC-AUC, classification of functional pathways, immune cell deconvolution results, and validation techniques of identified features.

### 2.8. Risk of Bias and Evidence Certainty

The methodological quality of included studies was assessed using an adapted version of the Critical Appraisal Skills Programme (CASP) checklist tailored to bioinformatic and transcriptomic analyses [13]. The full adapted checklist is provided in Supplementary Table S2. The checklist evaluated the appropriateness of dataset usage, analytic strategy transparency, reproducibility of methods, and validation robustness. In addition, the GRADE-CERQual framework was applied to appraise confidence in the findings emerging from the functional meta-synthesis, considering domains such as methodological limitations, coherence, adequacy, and relevance [14].

### 2.9. Data Synthesis and Functional Meta-Synthesis

A functional meta-synthesis approach was employed to thematically integrate findings from bioinformatic and transcriptomic studies. Narrative synthesis was used to categorize and map biomarker candidates, immune cell infiltrates, and microbial profiles. Thematic coding was applied to group findings into domains such as immune signaling, fibroblast activity, microbiome dysbiosis, and diagnostic modeling. A synthesis matrix was constructed to compare results across studies, and a network visualization was developed to illustrate interrelationships among key biomarkers and pathways.

### 2.10. Diagnostic Meta-Analysis

When three or more studies reported comparable ROC-AUC values from predictive machine learning models, a diagnostic meta-analysis was conducted. Logit-transformed AUC values were pooled using a random-effects model with the DerSimonian–Laird estimator. Heterogeneity was assessed using the I^2^ and τ^2^ statistics. Subgroup analyses were performed based on biomarker category (e.g., transcriptomic, immune, microbial) and type of computational model. All statistical analyses were conducted using Python 3.11 and relevant open-source packages including Statsmodels v0.14.1, SciPy v1.11.4, Pandas v2.1.4, and Matplotlib v3.8.2.

## 3. Results

### 3.1. Study Selection

**Figure 1:**
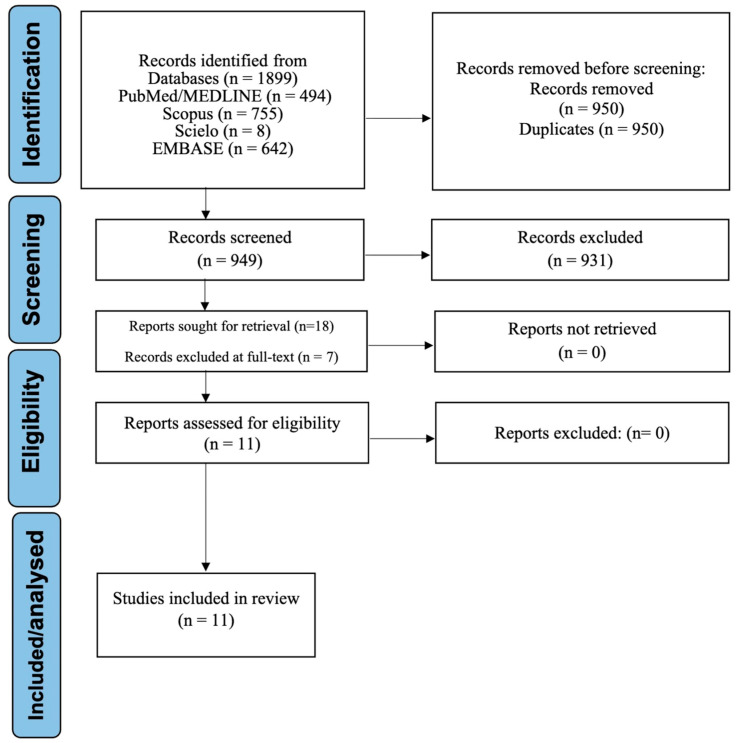
PRISMA flowchart depicting the study selection process for the functional meta-synthesis and diagnostic meta-analysis.

A total of 1899 records were retrieved through systematic searches across PubMed, EMBASE, SciELO, and Scopus. After removal of 950 duplicates, 949 unique records remained for title and abstract screening. Of these, 931 were excluded for irrelevance (e.g., in vitro studies, animal models, non-bioinformatic approaches, or unrelated oral conditions). Eighteen full-text articles were then assessed for eligibility. Seven of these were excluded due to a lack of human tissue samples, absence of machine learning or transcriptomic analysis, or failure to report biomarker-related outcomes. Ultimately, 11 studies met all inclusion criteria and were incorporated into the final synthesis [15,16,17,18,19,20,21,22,23,24,25]. The complete selection process is detailed in Figure 1. In accordance with PRISMA 2020 [12], Supplementary Table S3 lists the seven full-text articles excluded at the eligibility stage, together with explicit reasons for their exclusion.

### 3.2. Overview of Included Studies

This systematic review, structured as a functional meta-synthesis and diagnostic meta-analysis, integrates findings from studies published between 2020 and 2025. These investigations employed diverse omics-based approaches—primarily transcriptomics, metagenomics, and integrative bioinformatics—to explore biomarkers and immune-microbial signatures associated with peri-implantitis. Most utilized human-derived peri-implant or gingival tissue samples. Advanced machine learning techniques such as LASSO, SVM-RFE, Boruta, and ensemble models were frequently used for biomarker selection and validation. Sample sizes ranged from small pilot datasets to moderate cohorts. The included studies employed internal validation, cross-validation, or external validation datasets to assess the robustness of their findings. Reported diagnostic performance metrics, when available, showed high accuracy, with AUC values often exceeding 0.88. Table 1 summarizes the core methodological and analytical characteristics of the included studies.

### 3.3. Functional Meta-Synthesis of Biomarkers and Pathways

**Figure 2:**
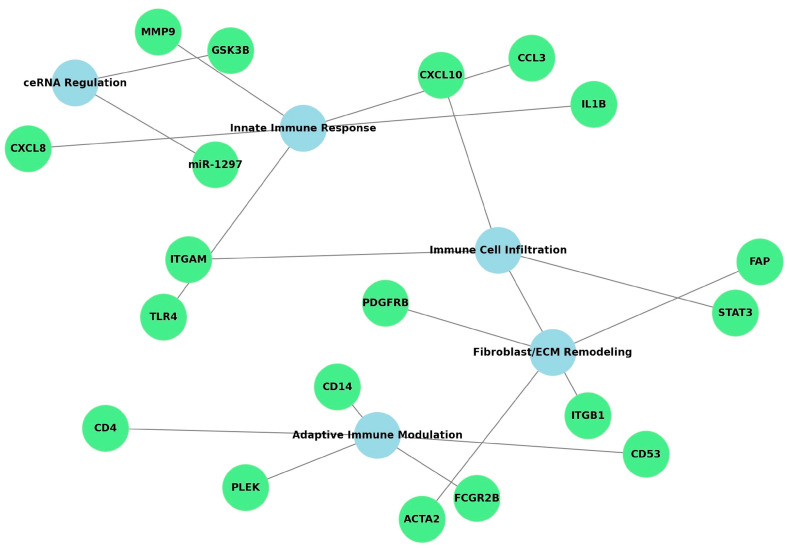
Network map showing the relationships among five biomarker classes identified through functional meta-synthesis: innate and adaptive immune responses, immune cell infiltration, fibroblast activation, and ceRNA regulatory networks. Key gene nodes (e.g., *IL1B*, *TLR4*, *ACTA2*, *GSK3B*) are color-coded by functional category and connected to major signaling pathways (e.g., TLR, PI3K-Akt, Wnt).

A functional meta-synthesis across all included studies revealed five dominant biological themes involved in peri-implantitis: innate immune response, adaptive immune modulation, immune cell infiltration, fibroblast activation and extracellular matrix (ECM) remodeling, and competing endogenous RNA (ceRNA) regulatory networks. Innate immune markers such as *IL1B*, *TLR4*, *CXCL8*, and *MMP9* were consistently overexpressed and enriched in cytokine-cytokine receptor interactions, Toll-like receptor (TLR), and NOD-like receptor signaling pathways. Adaptive immune regulation was marked by dysregulation of *CD4*, *CD14*, *FCGR2B* and intersected with NF-κB and osteoclast differentiation pathways. Immune infiltration, particularly involving dendritic cells and NK-T cells, correlated with *STAT3*, *CXCL10*, and other markers of inflammation. Fibroblast-related markers such as *ACTA2*, *FAP*, and *PDGFRB*, described by Oh et al. [17], reflected ECM remodeling mechanisms. Lastly, Li et al. [24] reported a ceRNA immunogenomic axis involving *GSK3B* and *miR-1297*. The network visualization (Figure 2) highlights the interconnected nature of these biological themes, revealing shared regulatory pathways and clustering of functionally related gene modules. This reinforces the multifactorial and integrative biological processes driving peri-implantitis.

The detailed functional classification of the five core biomarker themes—based on contributing studies, representative genes, and enriched pathways—is summarized in Table 2. This matrix complements the network visualization by organizing the evidence thematically and supporting the synthesis of immuno-genomic signatures in peri-implantitis.

### 3.4. Diagnostic Meta-Analysis of Machine Learning Models

**Figure 3:**
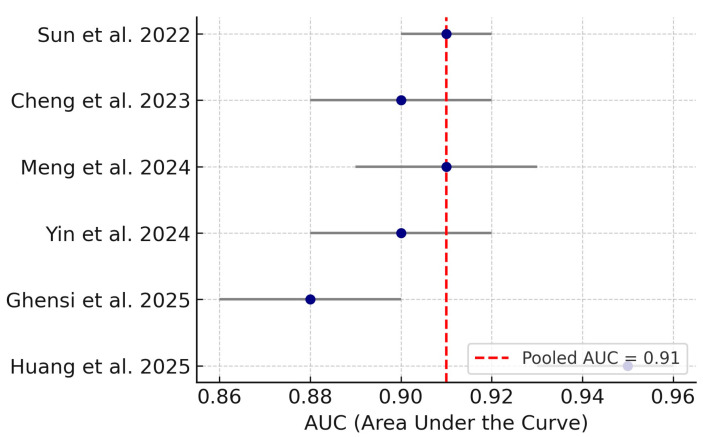
Forest plot of ROC-AUC values for ML-based models predicting peri-implantitis. Pooled AUC = 0.91 (95% CI: 0.88–0.93). Data derived from Sun et al. 2022 [22], Cheng et al. 2023 [21], Meng et al. 2024 [19], Yin et al. 2024 [18], Ghensi et al. 2025 [16], and Huang et al. 2025 [15].

A random-effects meta-analysis was performed on ROC-AUC values reported in six eligible studies using transcriptomic, genomic, and metagenomic data. Genomic biomarkers showed AUCs between 0.92 and 0.95 [15,19]. Transcriptomic biomarkers achieved AUCs from 0.89 to 0.91 [18,21,22]. The metagenomic-based classifier reported an AUC of 0.88 [16]. The combined pooled estimate across studies was 0.91 (95% CI: 0.88–0.93), with I^2^ = 0%, indicating no heterogeneity (I^2^ = 0%, τ^2^ ≈ 0). This suggests robust and generalizable diagnostic performance of ML-integrated biomarker strategies (Figure 3).

**Figure 4:**
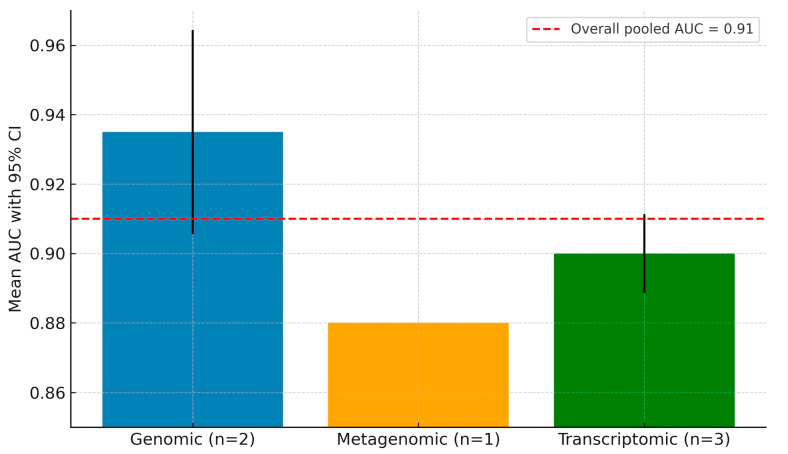
Subgroup AUC performance for genomic, transcriptomic, and metagenomic models. Overall pooled AUC = 0.91.

To further explore diagnostic variability by biomarker class, a subgroup meta-analysis was conducted. Figure 4 presents a comparative bar chart of AUC performance by biomarker subgroup. Genomic and transcriptomic biomarkers both demonstrated high and consistent AUC values (>0.90), while metagenomic models showed slightly lower pooled performance with wider confidence intervals. These results underscore the stronger discriminative potential of host-derived molecular signatures in peri-implantitis classification tasks.

### 3.5. Additional Functional Insights and Secondary Outcomes

Beyond AUC metrics, several studies incorporated immune cell deconvolution techniques (e.g., ssGSEA, CIBERSORT) to map immune microenvironment profiles. Cheng et al. [21] and Chen et al. [20] identified altered proportions of dendritic cells, macrophages, and immature B cells in peri-implant tissues. Other validation strategies included qPCR, PPI network reconstruction, and immunohistochemistry [18,20], reinforcing the functional relevance of bioinformatic predictions. These results support the translational potential of molecular and immune biomarkers in refining peri-implantitis diagnosis and pathophysiological understanding.

### 3.6. Risk of Bias and Evidence Certainty

The methodological quality of the included studies was assessed using an adapted version of the CASP checklist for bioinformatic and transcriptomic research [14]. Most studies demonstrated moderate to high methodological rigor, with comprehensive descriptions of sample handling and analytical approaches. Some studies did not include external validation or qPCR confirmation, which may reflect differences in design scope rather than methodological limitations. Reporting transparency also showed some variability (Table 3).

Certainty of evidence from the functional meta-synthesis was evaluated using the GRADE-CERQual approach [15]. Most biological themes—such as innate immune response and adaptive immune modulation—were rated as having high overall confidence, supported by minor methodological limitations, high coherence among studies, and high adequacy of data. The theme of immune cell infiltration received a moderate confidence rating, reflecting moderate levels across methodological limitations, coherence, and adequacy domains. In contrast, the themes of fibroblast activation with extracellular matrix remodeling and ceRNA network regulation were assigned lower overall confidence, largely reflecting the fact that available evidence originated from a limited number of studies, with some methodological constraints and lower consistency across findings.

## 4. Discussion

This systematic review combined a functional meta-synthesis and a diagnostic meta-analysis to explore the molecular and predictive landscape of peri-implantitis through transcriptomic and bioinformatic evidence. Eleven studies published between 2020 and 2025 were included, with most applying transcriptomic, immune-infiltration, or integrative omics methods to analyze peri-implant tissues. These studies utilized human-derived gingival or peri-implant tissues to identify immune and stromal biomarkers, signaling pathways, and potential therapeutic targets. Several employed machine learning techniques such as LASSO, SVM-RFE, and Boruta for biomarker selection and model validation. Diagnostic accuracy, when assessed, showed promising AUC values, frequently exceeding 0.88, highlighting the potential of omics-guided models to inform clinical diagnosis.

The functional meta-synthesis revealed five recurring biological themes in peri-implantitis: innate and adaptive immune activation, immune cell infiltration, fibroblast-driven ECM remodeling, and ceRNA regulatory networks. Studies like Li et al. [21], Chen et al. [20], and Oh et al. [17] consistently reported upregulation of *IL1B*, *TLR4*, *CXCL8*, and *MMP9* in peri-implantitis, aligning with heightened Toll-like receptor and cytokine-mediated signaling. These findings were reinforced by pathway enrichment analyses identifying involvement of NF-κB, osteoclast differentiation, and PI3K-Akt cascades [24,26,27]. Other investigators, such as Yin et al. [18], also described elevated adaptive immune signatures, particularly involving *CD4+* T cells and transcription factors like *FOXP3* and *RORγt*. Importantly, these patterns echo findings from related inflammatory oral diseases and systemic conditions like rheumatoid arthritis, lending support to shared immunopathogenic mechanisms [24,28,29].

The diagnostic meta-analysis demonstrated that machine learning-based models leveraging transcriptomic data can achieve high diagnostic performance. Studies by Huang et al. [15] and Meng et al. [19] reported AUC values above 0.90, validating their models with either cross-validation or independent external datasets. In particular, Huang et al. [15] identified *TLR4* and *FLT3* as robust classifiers of peri-implantitis, further confirmed through qPCR and ROC curve analysis using GSE223924. Conversely, Li et al. [21] and Sun et al. [22] used SVM and RFE algorithms without full external validation, a limitation observed across several studies. These findings support the potential of integrating omics and ML tools in diagnostic workflows, although standardization and validation remain necessary [30,31,32]. Future studies should prioritize external validation using independent cohorts and prospective designs to ensure robustness and reproducibility.

Beyond primary immune signals, other functional axes were identified. Oh et al. [17] highlighted fibroblast activation and matrix remodeling via *ACTA2*, *FAP*, and *PDGFRB*, linking structural tissue changes to disease progression. Similarly, Li et al. [25] reported ceRNA interactions involving *GSK3B* and *miR-1297*, suggesting epigenomic regulation in peri-implant inflammation. These secondary findings reveal peri-implantitis as a multifactorial disease with both inflammatory and stromal components. Comparable ceRNA patterns have been implicated in oral squamous cell carcinoma and autoimmune conditions, suggesting that similar post-transcriptional mechanisms may be operative [33,34,35].

Risk of bias assessment using an adapted CASP checklist revealed moderate to high methodological rigor across studies, especially in data preprocessing and statistical modeling. However, only a subset of studies—namely, Huang et al. [15] and Meng et al. [19]—included external validation or qPCR confirmation. Others lacked transparency in dataset integration or preprocessing protocols. According to GRADE-CERQual, confidence in the functional synthesis was rated as moderate, primarily due to inconsistency in validation and selective reporting of performance metrics. This aligns with prior critiques of bioinformatics analyses, which emphasized the importance of multi-cohort validation to ensure reproducibility [36,37].

An important translational consideration involves the feasibility of obtaining biological specimens for biomarker analysis. While peri-implant or gingival tissue biopsies remain the most informative for transcriptomic profiling [38], their invasive nature limits routine applicability. However, less invasive approaches—such as sampling of peri-implant crevicular fluid or saliva—are increasingly feasible and have been shown to capture several of the immune and molecular markers identified in this review [39]. These approaches may enhance patient acceptance and reduce clinical risk. From an economic perspective, high-throughput sequencing and advanced bioinformatics pipelines currently entail higher costs than conventional diagnostics [40]. Nonetheless, progressive reductions in sequencing costs and the development of targeted, multiplex assays suggest that biomarker-based diagnostics may become economically viable in specialized or high-risk populations [41]. Taken together, while translational hurdles remain, the clinical applicability of non-invasive sampling combined with machine learning models holds considerable promise for peri-implant disease management.

Clinically, the findings underscore the potential of transcriptomic and machine learning integration for early and non-invasive diagnosis of peri-implantitis. Key biomarkers like *IL1B*, *TLR4*, *CXCL8*, and *ACTA2* may guide salivary or crevicular fluid tests, while ML-based models could complement clinical indices and radiographic findings. The identified gene signatures and immune pathways also offer therapeutic insight, suggesting targets for anti-inflammatory or anti-fibrotic interventions. For example, the identification of *TLR4* and *FLT3* could support future drug repurposing trials using *TLR* or kinase inhibitors [42,43,44].

While this review offers comprehensive insights, certain considerations should be acknowledged. Variations in study designs, sample sizes, and datasets may have influenced the comparability of some findings, although the core biological themes and diagnostic trends remained consistent across investigations. In several cases, methodological details—such as batch correction, preprocessing steps, or platform normalization—were not fully described, which may reflect differences in reporting rather than analytical rigor. Moreover, although a number of studies incorporated predictive models, external validation and wet-lab confirmation were not universally performed; nevertheless, the convergence of biomarker profiles and diagnostic metrics across multiple datasets supports the robustness of the overarching conclusions.

The geographic concentration of evidence represents an important constraint: most of the included studies were conducted in Asian populations, primarily in China, with only a small number from Europe and South Korea. Although the identified transcriptomic and immunological biomarkers reflect fundamental biological processes that are not restricted to a specific ethnic or cultural background [45], the predominance of Asian cohorts limits the immediate generalizability of findings to other regions. Future multicenter investigations including European, American, and more diverse populations are needed to confirm the universality and clinical applicability of these biomarkers.

Publication bias may also be present, as gray literature sources (e.g., theses, conference proceedings, trial registries) were not systematically searched. Although the strategy encompassed four major international databases to maximize the retrieval of peer-reviewed evidence, the omission of gray literature may have reduced the likelihood of identifying studies with negative or inconclusive findings.

Finally, the scope of this review was deliberately limited to transcriptomic, metagenomic, and bioinformatic biomarkers. Other relevant omics layers, such as proteomics, metabolomics, or epigenomics, were beyond the protocol and therefore not included. Consequently, the conclusions drawn here cannot be generalized to all omic-derived biomarkers, and further systematic reviews are warranted to explore these additional molecular domains. A further methodological issue relates to the risk of overfitting in the primary machine learning models. Most included studies relied on internal validation strategies such as cross-validation or train/test splits, which, while useful for model optimization, do not fully account for generalizability. Only a minority of studies employed true external validation using independent cohorts. This imbalance underscores the need for future research to incorporate rigorous external validation frameworks across multi-center and heterogeneous populations to ensure reproducibility and reduce the risk of model overfitting.

Despite these limitations, this review is the first to combine functional meta-synthesis with diagnostic meta-analysis to comprehensively characterize peri-implantitis biomarkers. The inclusion of human peri-implant samples, application of multiple machine learning algorithms, and transparent synthesis of omics-level findings support the scientific validity of the conclusions. This integrative approach highlights both the biological complexity and the translational promise of using bioinformatics and AI to tackle peri-implant diseases.

## 5. Conclusions

The integration of transcriptomic, metagenomic, and bioinformatic data with advanced computational tools, including machine learning-based feature selection, can improve the precision of peri-implantitis diagnosis beyond traditional clinical and radiographic assessments. Transcriptomic profiling, immune cell deconvolution, and bioinformatics-driven biomarker identification enable earlier detection, more accurate risk stratification, and the development of diagnostic tools to complement current clinical practice. Key biomarkers identified in this review—such as IL1B, TLR4, CXCL8, ACTA2, and GSK3B—offer translational potential not only for diagnosis but also for guiding targeted therapeutic strategies, including anti-inflammatory and anti-fibrotic interventions, reinforcing their clinical relevance. These approaches may help clinicians adopt a more personalized and mechanistically informed management of peri-implant diseases.

Future investigations should prioritize methodological transparency, standardized validation pipelines, and multi-cohort prospective designs to ensure the reproducibility and clinical applicability of computationally derived biomarkers. External validation using independent datasets, combined with wet-lab confirmation (e.g., qPCR and immunohistochemistry), is essential to confirm diagnostic performance and functional relevance. Furthermore, exploring the therapeutic modulation of pathways such as TLR4- and FLT3-mediated signaling will be crucial, together with assessing the cost-effectiveness and feasibility of incorporating molecular diagnostics into peri-implant care. Cross-disciplinary collaboration between clinicians, bioinformaticians, and translational scientists will be critical to fully realize the potential of bioinformatics and AI in precision peri-implant care. It should also be noted that the present review was limited to transcriptomic, metagenomic, and bioinformatic biomarkers; therefore, the conclusions cannot be extrapolated to other omics domains such as proteomics, metabolomics, or epigenomics.

## Figures and Tables

**Table 1:** Core Characteristics of Included Studies.

Author (Year)	Country	Design	Omics Type	Sample Type	Biomarker Type	ML/Bioinfo Method	Diagnostic Metric
Huang et al. (2025) [15]	China	Transcriptomic ML	Microarray	Peri-implant tissue	Ferroptosis, immune	LASSO, SVM-RFE, Boruta	AUC: 0.95
Ghensi et al. (2025) [16]	Italy	Metagenomic	Shotgun metagenomics	Submucosal plaque	Microbial	Taxonomic/functional + ML	AUC: 0.88
Oh et al. (2024) [17]	South Korea	Transcriptomic	RNA-seq	PI vs. Periodontitis tissue	Fibroblast markers	DEG analysis	Not reported
Yin et al. (2024) [18]	China	Transcriptomic	Microarray	Peri-implant tissue	Adaptive immune genes	Enrichment, ROC, qPCR	AUC: 0.90
Meng et al. (2024) [19]	China	Transcriptomic ML	Microarray	Gingival tissue	Immune genes	LASSO, SVM-RFE, qPCR	AUC: 0.92
Chen et al. (2024) [20]	China	Transcriptomic	Microarray	PI vs. healthy gingiva	Immune + DEG	PPI, enrichment, IHC	Not reported
Cheng et al. (2023) [21]	China	Transcriptomic	RNA-seq	Peri-implant tissues	Immune cell profiles	WGCNA, ssGSEA	AUC: 0.89
Sun et al. (2022) [22]	China	Transcriptomic ML	Microarray	Peri-implant tissue	DEG + function	ML modeling, enrichment	AUC: 0.91
Zhang et al. (2021) [23]	China	Transcriptomic	RNA-seq	Peri-implant tissue	Immune hub genes	WGCNA, clustering	Not reported
Li J et al. (2021) [24]	China	Transcriptomic ML	Microarray	Peri-implant tissues	Diagnostic DEGs	Feature selection, ML	AUC: 0.89
Li M et al. (2020) [25]	China	Transcriptomic	Microarray	Peri-implant gingiva	Immune + ceRNA	ceRNA, DEG analysis	Not reported

DEG = Differentially Expressed Gene; ML = Machine Learning; RNA-seq = RNA sequencing; PPI = Protein–Protein Interaction; IHC = Immunohistochemistry; qPCR = quantitative Polymerase Chain Reaction; LASSO = Least Absolute Shrinkage and Selection Operator; SVM-RFE = Support Vector Machine–Recursive Feature Elimination; WGCNA = Weighted Gene Co-expression Network Analysis; ssGSEA = single-sample Gene Set Enrichment Analysis; ceRNA = competing endogenous RNA; AUC = Area Under the Curve.

**Table 2:** Functional Biological Themes Identified Across Studies.

Biological Theme	Contributing Studies	Key Genes	Associated Pathways
Innate Immune Response	Chen et al. [20], Cheng et al. [21], Zhang et al. [23]	*IL1B*, *TLR4*, *CXCL8*, *CCL3*, *MMP9*	Cytokine-cytokine receptor interaction, TLR signaling
Adaptive Immune Modulation	Yin et al. [18], Li et al. [24], Chen et al. [20]	*CD4*, *CD14*, *FCGR2B*, *CD53*, *PLEK*	T cell receptor, NF-κB, Osteoclast differentiation
Immune Cell Infiltration	Chen et al. [20], Li et al. [25], Cheng et al. [21]	*ITGAM*, *STAT3*, *CXCL10*	Chemokine signaling, Leukocyte migration
Fibroblast Activation and ECM	Oh et al. [17]	*ACTA2*, *FAP*, *PDGFRB*	PI3K-Akt, ECM-receptor interaction
ceRNA Networks	Li et al. [25]	*GSK3B*, *miR-1297*	ceRNA regulation, Wnt signaling

ceRNA = competing endogenous RNA; ECM = extracellular matrix; TLR = Toll-like receptor; PI3K-Akt = phosphatidylinositol 3-kinase/protein kinase B; NF-κB = nuclear factor kappa-light-chain-enhancer of activated B cells; Wnt = wingless-related integration site.

**Table 3:** CASP Quality Appraisal of Included Studies.

Study	Clear Aim and Design	Data Appropriateness	Analytic Transparency	Validation Robustness
Huang et al. [15]	Yes	Yes	Yes	Yes
Ghensi et al. [16]	Yes	Yes	Yes	Moderate
Oh et al. [17]	Yes	Yes	Moderate	No
Yin et al. [18]	Yes	Yes	Yes	Yes
Meng et al. [19]	Yes	Yes	Yes	Yes
Chen et al. [20]	Yes	Yes	Moderate	Moderate
Cheng et al. [21]	Yes	Yes	Yes	Moderate
Sun et al. [22]	Yes	Yes	Yes	Yes
Zhang et al. [23]	Yes	Moderate	Moderate	No
Li J et al. [24]	Yes	Yes	Yes	Moderate
Li M et al. [25]	Yes	Moderate	Moderate	No

## Data Availability

The original contributions presented in this study are included in the article/Supplementary Material. Further inquiries can be directed to the corresponding author.

## Supplementary Materials

The following supporting information can be downloaded at: https://www.mdpi.com/article/10.3390/medsci13030187/s1, Table S1. Complete Search Strategies for Each Database. Table S2. Adapted CASP checklist for risk of bias assessment. Table S3. Full-text articles excluded at the eligibility stage with reasons.
