# Supplementary material for: Transcriptomic and Metagenomic Biomarkers in Peri-Implantitis: A Systematic Review, Diagnostic Meta-Analysis, and Functional Meta-Synthesis
Source: Med Sci (Basel). 2025 Sep 12;13(3):187. doi: 10.3390/medsci13030187 (PMC12452457; doi:10.3390/medsci13030187)
Supplement: Supplementary file 1 [file medsci-13-00187-s001.zip › Table S1.pdf]

Supplementary Table S1. Database-specific search strategies

Scope and limits: Searches were designed to combine controlled vocabulary with free-text terms and were run with no language restrictions. Results should be limited to publications up to July 31, 2025 (no lower date limit). Where available, exclude animal-only records. These strings are copy/paste-ready; apply date limits via each platform's filters to match the study's cutoff.

Database (Interface)	Exact query	Filters / Limits to apply
PubMed/MEDLINE	(("Peri-Implantitis"[Mesh] OR peri-implantit*[tiab] OR periimplantit*[tiab]) AND ("Computational Biology"[Mesh] OR bioinformatics[tiab] OR "Machine Learning"[Mesh] OR "Artificial Intelligence"[Mesh] OR "Transcriptome"[Mesh] OR "Gene Expression Profiling"[Mesh] OR "RNA-Seq"[tiab] OR "Metagenomics"[Mesh] OR "Biomarkers"[Mesh] OR "Microbiota"[Mesh] OR WGCNA[tiab] OR "weighted gene co-expression net*"[tiab] OR ceRNA[tiab] OR "gene expression"[tiab] OR transcriptom*[tiab] OR metagenom*[tiab] OR microbiom*[tiab] OR microbiota[tiab] OR "immune signature*"[tiab] OR "differential expression"[tiab] OR CIBERSORT[tiab] OR deconvolution[tiab])) NOT (animals[MeSH Terms] NOT humans[MeSH Terms])	Publication date: up to 2025-07-31; No language limits; Exclude animal-only: NOT (animals[MeSH] NOT humans[MeSH])
Embase (Ovid/Elsevier)	(('periimplantitis'/exp OR peri-implantit*:ti,ab,kw OR periimplantit*:ti,ab,kw) AND ('computational biology'/exp OR	Limit to humans; Publication year:

	bioinformatics:ti,ab,kw OR 'machine learning'/exp OR 'artificial intelligence'/exp OR 'transcriptome'/exp OR 'gene expression profiling'/exp OR 'rna sequencing'/exp OR 'metagenomics'/exp OR 'biomarker'/exp OR microbiota/exp OR (RNA-Seq OR WGCNA OR "weighted gene co-expression" OR ceRNA OR "gene expression" OR transcriptom* OR metagenom* OR microbiom* OR microbiota OR "immune signature*" OR "differential expression" OR CIBERSORT OR deconvolution):ti,ab,kw)) NOT ([animals]/lim NOT [humans]/lim)	up to 2025-07-31; No language limits
Scopus (Elsevier)	TITLE-ABS-KEY (("peri-implantitis" OR periimplantitis) AND (bioinformatics OR "machine learning" OR "artificial intelligence" OR "RNA-Seq" OR "gene expression" OR transcriptom* OR WGCNA OR "weighted gene co-expression" OR ceRNA OR metagenom* OR biomarker* OR microbiom* OR microbiota OR "immune signature" OR "differential expression" OR CIBERSORT OR deconvolution))	Refine results to publication year ≤ 2025 and to Articles/Reviews only if desired; No language limits
SciELO	("peri-implantitis" OR periimplantitis OR "peri-implantite" OR periimplantite) AND (bioinformatics OR "machine learning" OR "artificial intelligence" OR "inteligencia artificial" OR "inteligência artificial" OR "RNA-Seq" OR "gene expression" OR "expresión génica" OR "expressão gênica" OR transcriptom* OR WGCNA OR "coexpresión génica"	No language limits; apply publication year ≤ 2025; use Advanced Search with Boolean operators

	OR "coexpressão gênica" OR ceRNA OR metagenom* OR "metagenómica" OR "metagenômica" OR biomarcador* OR "immune signature" OR "firma inmune" OR "assinatura imune" OR microbiom* OR microbiota)

Notes:

• Embase syntax shown here matches Ovid. In embase.com, use field tags :ti,ab,kw similarly. • In PubMed, 'Computational Biology'[Mesh] was used instead of 'Bioinformatics' since the latter is not a valid MeSH. • Consider adding specific ML algorithm keywords (e.g., random forest, SVM, LASSO) if increased recall is needed. • Reference lists of included studies were manually screened to identify additional records.
