# Supplementary material for: Transcriptomic and Metagenomic Biomarkers in Peri-Implantitis: A Systematic Review, Diagnostic Meta-Analysis, and Functional Meta-Synthesis
Source: Med Sci (Basel). 2025 Sep 12;13(3):187. doi: 10.3390/medsci13030187 (PMC12452457; doi:10.3390/medsci13030187)
Supplement: Supplementary file 1 [file medsci-13-00187-s001.zip › Table S2.pdf]

Supplementary Table S2. Adapted CASP checklist for risk of bias assessment

Domain	Item	Guiding question / Criterion
Study validity	Clear research question	Was the study objective clearly stated and focused on peri-implant biomarkers?
Study validity	Appropriate study design	Was the study design suitable to address the research question (observational, case-control, cohort)?
Study validity	Eligibility criteria	Were inclusion and exclusion criteria explicitly described?
Biomarker assessment	Measurement validity	Were biomarker measurements clearly described and validated (e.g., RNA-seq, metagenomics)?
Biomarker assessment	Sample source	Were samples derived from human tissue, saliva, or peri-implant crevicular fluid?
Biomarker assessment	Quality control	Was there evidence of adequate preprocessing and quality control of omics data?
Machine learning methodology	Model description	Was the machine learning algorithm clearly described (e.g., type, parameters)?
Machine learning methodology	Feature selection	Was feature selection or dimensionality reduction performed appropriately?
Machine learning methodology	Validation strategy	Was internal validation (cross-validation, split-sample) performed? Was external validation reported?
Machine learning methodology	Overfitting prevention	Were strategies to prevent overfitting described (regularization, independent test sets)?
Reporting transparency	Outcome reporting	Were model performance metrics (AUC, sensitivity, specificity) reported with confidence intervals?
Reporting transparency	Reproducibility	Was there sufficient methodological detail to allow replication (data availability, code, workflow)?

Overall assessment	Risk of bias judgment	Based on the above items, is the risk of bias low, unclear, or high?
