# Supplementary material for: Transcriptomic and Metagenomic Biomarkers in Peri-Implantitis: A Systematic Review, Diagnostic Meta-Analysis, and Functional Meta-Synthesis
Source: Med Sci (Basel). 2025 Sep 12;13(3):187. doi: 10.3390/medsci13030187 (PMC12452457; doi:10.3390/medsci13030187)
Supplement: Supplementary file 1 [file medsci-13-00187-s001.zip › Table S3 .pdf]

Supplementary Table S3. Full-text articles excluded at the eligibility stage with reasons

Reference	Title / Journal	Reason for Exclusion
de Araújo Silva DN, Casarin M, Monajemzadeh S, Menezes da Silveira T, Lubben J, Bezerra B, et al. Experimental Model of Ligature-Induced Peri-Implantitis in Mice. J Vis Exp. 2024 May 17;(207). doi:10.3791/66316.	Experimental Model of Ligature-Induced Peri-Implantitis in Mice (J Vis Exp)	Lack of human tissue samples (murine model)
Tong ZA, Chen L, Shen L, Lu YF, Zhang JW, Qi YD, et al. A Modified Ligature-Induced Peri-Implantitis Murine Model and RNA Sequencing Analysis Compared With Human Subjects. J Clin Periodontol. 2025 May;52(5):787-798. doi:10.1111/jcpe.14127.	A Modified Ligature-Induced Peri-Implantitis Murine Model and RNA Sequencing Analysis Compared With Human Subjects (J Clin Periodontol)	Lack of human tissue samples (murine model)
Wang Q, Haugen HJ, Linke D, Lyngstadaas SP, Sigurjónsson ÓE, Ma Q. Impact of different chemical debridement agents on early cellular responses to titanium dental implants: A transcriptome-based in vitro study on peri-implant tissue regeneration. Colloids Surf B Biointerfaces. 2025 Sep;253:114727. doi:10.1016/j.colsurfb.2025.114727.	Impact of different chemical debridement agents on early cellular responses to titanium dental implants: A transcriptome-based in vitro study on peri-implant tissue regeneration. (Colloids Surf B Biointerfaces)	Lack of human tissue samples (in vitro cell model)
Liu X, Deng S, Li X, Liu H, Li Z, Wu Y, et al. A Standardized Rat Model to Study Peri-implantitis of Transmucosal Osseointegrated Implants. Biomater Res. 2024 Jun 1;28:0021. doi:10.34133/bmr.0021.	A Standardized Rat Model to Study Peri-implantitis of Transmucosal Osseointegrated	Lack of human tissue samples (rat model)

	Implants. (Biomater Res)	
Cafferata EA, Ramanauskaite A, Cuypers A, Obreja K, Dohle E, Ghanaati S, et al. Experimental peri-implantitis induces neuroinflammation: An exploratory study in rats. BMC Oral Health. 2024 Oct 18;24(1):1238. doi:10.1186/s12903-024-04995-z.	Experimental peri-implantitis induces neuroinflammation: An exploratory study in rats (BMC Oral Health)	Lack of human tissue samples (rat model)
Hentenaar DFM, de Waal YCM, Vissink A, van Winkelhoff AJ, Meijer HJA, Liefers SC, et al. Biomarker levels in peri-implant crevicular fluid of healthy implants, untreated and non-surgically treated implants with peri-implantitis. J Clin Periodontol. 2021 Apr;48(4):590-601. doi:10.1111/jcpe.13423.	Biomarker levels in peri-implant crevicular fluid of healthy implants, untreated and non-surgically treated implants with peri-implantitis. (J Clin Periodontol)	Absence of machine learning or transcriptomic analysis
Awad AI, Abou Khadr MA, Abdelhady S, Abdelaziz R. Evaluation of endocan biomarker levels in peri-implant crevicular fluid of healthy and diseased peri-implant sites: a cross-sectional study. BMC Oral Health. 2025 Jul 30;25(1):1276. doi:10.1186/s12903-025-06633-8.	Evaluation of endocan biomarker levels in peri-implant crevicular fluid of healthy and diseased peri-implant sites: a cross-sectional study (BMC Oral Health)	Absence of machine learning or transcriptomic analysis
